# Biomechanical enhancement in rotator cuff repairs: the impact of innovative nanofiber technology

**DOI:** 10.1016/j.jseint.2024.08.203

**Published:** 2024-09-10

**Authors:** James Johnson, Ben Gadomski, Daniel Regan, Jed Johnson, Brad Nelson, Kirk McGilvray, Kevin Labus, Anthony Romeo, Jeremiah Easley

**Affiliations:** a Orthopedic Bioengineering Research Laboratory, Colorado State University, Fort Collins, CO, USA; b Flint Animal Cancer Center and Department of Microbiology, Immunology, & Pathology, Fort Collins, CO, USA; c Atreon Orthopedics, Dublin, OH, USA; d Preclinical Surgical Research Laboratory, Colorado State University, Fort Collins, CO, USA; e USA State University, Fort Collins, CO, USA; f Rothman Orthopaedic Institute, New York, NY, USA

**Keywords:** Rotator cuff repair, Bioresorbable scaffold, Nanofiber technology, Tendon healing, Biomechanical enhancement, Tendon-bone interface

## Abstract

**Background:**

Rotator cuff repair surgeries often face high failure rates, particularly in cases involving tendon degeneration. Traditional repair techniques and devices frequently fail to adequately restore a healthy native enthesis and strong tendon-bone integration. This study investigates the efficacy of a novel, fully synthetic, bioresorbable nanofiber scaffold in restoring the native enthesis and enhancing the biomechanical properties and overall success of rotator cuff repairs, particularly in the context of chronically degenerated tendons.

**Methods:**

This study used an ovine model to simulate chronic tendon degeneration with subsequent rotator cuff transection and repair. All repairs were performed using the standard double-row configuration with suture tape; half of the repairs were augmented with the bioresorbable nanofiber scaffold. Nondestructive biomechanical testing was conducted to assess the strength of the repair constructs, followed by histological analysis of all tendon samples to evaluate tissue regeneration and integration at the repair site.

**Results:**

Results demonstrated that the scaffold group achieved significantly improved biomechanical properties (peak force, peak stress, equilibrium force, and equilibrium stress) compared to the suture only group, indicating enhanced repair strength and native enthesis restoration. Scaffold samples exhibited significantly decreased cross-sectional areas (ie, less fibrosis) which were similar to healthy tendons. Histological findings indicated the scaffold did not impede re-establishment of Sharpey-like fibers at the tendon insertion.

**Conclusion:**

This study provides compelling evidence that the use of a fully synthetic, bioresorbable nanofiber scaffold in rotator cuff repair significantly improves biomechanical outcomes and enthesis regeneration. These improvements were achieved while retaining close to native tendon thickness. The findings suggest that this scaffold represents a significant advancement in rotator cuff repair technology, offering a promising solution to enhance repair strength and quality of bone-tendon integration, especially in challenging cases of tendon degeneration.

The prevalence of rotator cuff tears has greatly increased over the years, with some estimates indicating a 3-fold increase between 1998 and 2011.^5^^,^^24^ The associated increase in surgical intervention prevalence reflects a growing awareness and diagnosis of these conditions; however, failures in these surgical outcomes persist at unacceptable levels, particularly for larger/massive tears.^12^^,^^20^^,^^37^ These failures can primarily be contributed to lack of re-establishment of the strong native fibrocartilaginous insertion at the tendon-bone interface.^6^^,^^7^ Thus, there remains a critical need to develop devices that not only augment these repairs but are also capable of re-establishing the native enthesis to improve repair outcomes.

In response to the ongoing challenges with rotator cuff repair outcomes, a novel bioabsorbable, interpositional nanofiber scaffold has been designed to augment the biologic environment in support of the healing cascade and remodeling of healthy tissue to improve long-term outcomes of rotator cuff repairs (Fig. 1). Its technology leverages a combination of polyglycolic acid and poly-L-lactide-co-ε-caprolactone polymers, creating a biphasic structure that supports the natural phases of healing and healthy tissue architecture remodeling.^10^^,^^11^^,^^23^ By leveraging the electrospinning process, this product is unique in that it has porosity and fiber diameters that mimic the extracellular matrix,^1^^,^^15^ providing a scaffold to promote cellular ingrowth and proliferation with the intent of increasing the postoperative strength of rotator cuff repair procedures.

**Figure 1:**
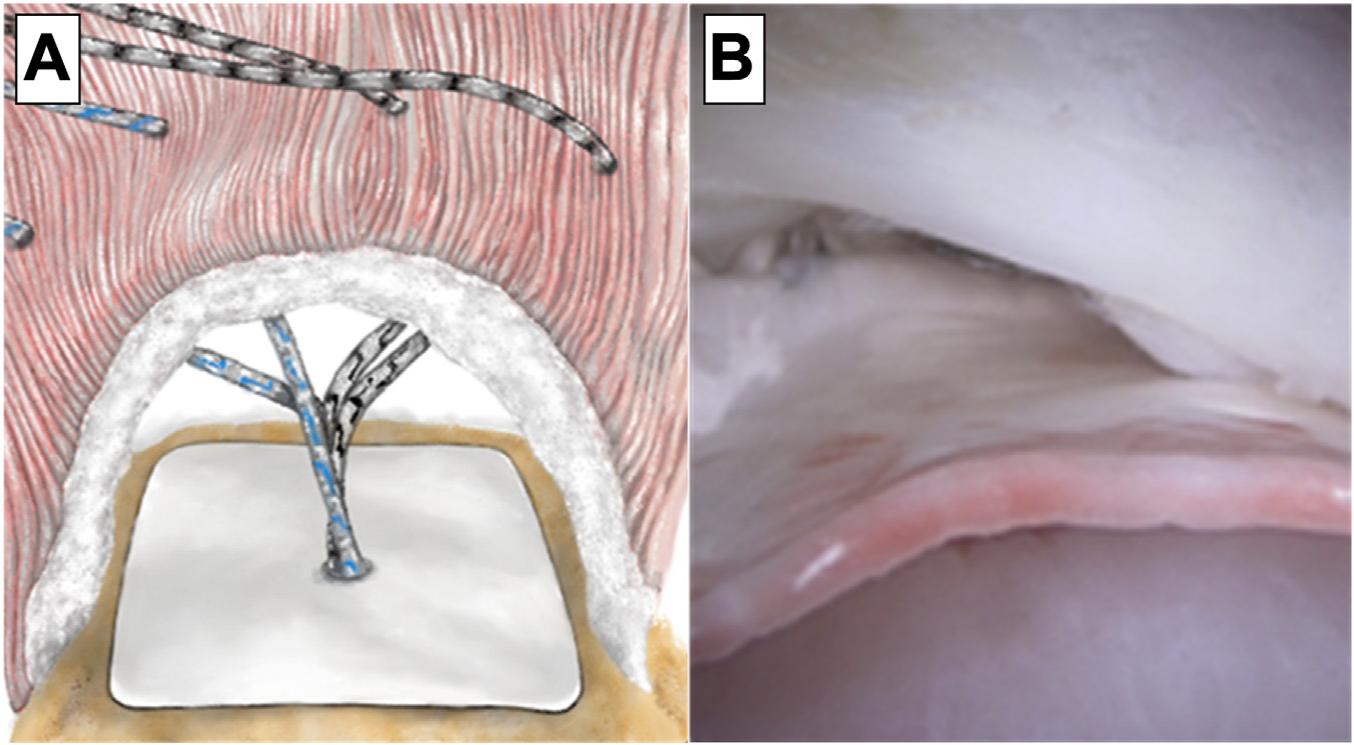
(**A**) Graphical illustration of the scaffold implanted in a representative human rotator cuff tear. (**B**) Intraoperative arthroscopic image of interpositional scaffold in human clinical case.

In contrast to other commercial offerings, the scaffold resorbs in 3-4 months and the degradants of this highly porous bioabsorbable scaffold offer significant benefits that contribute to its efficacy in tissue regeneration and healing. As the scaffold undergoes biphasic biodegradation, it releases a tailored dose of lactic acid, which plays a crucial role in modulating the local pH environment, promoting angiogenesis, and stimulating the migration of endothelial cells.^3^^,^^14^^,^^26^^,^^32^, ^33^^,^^35^ This localized acidification also enhances the antimicrobial properties of the scaffold.^2^^,^^25^ Additionally, the degradation products of the scaffold are involved in the modulation of macrophage activity, shifting them toward a pro-healing M2 phenotype, which is essential for tissue repair and regeneration.^13^^,^^31^ These bioactive degradants not only ensure the scaffold’s resorption but also actively participate in the healing process, differentiating this biphasic scaffold within the regenerative medicine category and collagen-based implants.

Improved healing using this device has been documented clinically,^4^^,^^30^ and increased ultimate strength has been noted in a nonchronic degeneration animal model^27^; however, the biomechanical strength of constructs using this scaffold in a chronically degenerated tendon model^16^ have not been reported. Chronic degeneration models, such as those previously developed by the authors,^8^^,^^16^, ^17^, ^18^ more closely replicate the challenging repair situations seen in the chronic massive tear situations. Specifically, these models have demonstrated decreased collagen organization, decreased bone quality underlying the insertion, decreased biomechanical properties, and increased fatty atrophy, all of which are hallmarks of chronic rotator cuff degeneration in humans. Challenging models like these provide a more realistic platform with which to assess device efficacy. As such, this study sought to characterize the biomechanical and histological benefits of augmenting rotator cuff repairs to chronically degenerated tendon tissue. It was hypothesized that augmentation of repairs with the interpositional scaffold would increase the biomechanical properties of constructs, while maintaining the biological ability to re-establish a fibrocartilaginous insertion (ie, Sharpey’s fibers).

## Methods

The study was conducted in accordance with the Declaration of Helsinki (as revised in 2013). Experiments were performed under a project license (No.: 18-7854A) granted by institutional committee board of Colorado State University, in compliance with US national or institutional guidelines for the care and use of animals.

### Surgical procedure to induce chronic degeneration

Unilateral surgical damage was created to the infraspinatus tendons in 8 skeletally mature (≥ 3.5 years of age) *Ovis aries* ewes resulting in n = 8 operated shoulders. Rams were not used in this study due to husbandry difficulties and aggressive behavior. The sheep were prepared for surgery and placed in left lateral recumbency under general anesthesia. A previously established surgical model (ie, sharp transection) was used to induce pathological changes akin to chronic degeneration.^16^ Briefly, standard surgical procedures were followed to visualize the superficial head and insertion of the infraspinatus tendon. With the ewe in lateral recumbency, an open approach was conducted to the level of the deltoid. After separation of fascial layer, the infraspinatus was visually identified under the deltoid, and the tendon insertion was located using palpation by a skilled surgeon. The enthesis damage model designed to replicate a large tear was accomplished via surgical insult accomplished by cutting 50% of the tendon fibers perpendicularly through the midportion of the attachment site on the humerus (ie, the footprint), replicative of a large tear. This model has been previously established and proven to induce chronic degeneration representative of what occurs in the human rotator cuff.^16^ Following induction of chronic degeneration, standard surgical closure procedures were followed. The sheep were allowed to eat and ambulate *ad libitum* during convalescence. Sheep were monitored daily throughout the study period for any signs of adverse events or complications and to evaluate pain, lameness/ambulatory function, and incisional site healing.

### Surgical repair procedure

Six weeks after the initial surgery that induced chronic degenerative changes in the sheep, a second procedure was performed that fully transected and repaired the tendons. Using the same surgical preparation and visualization procedures, the infraspinatus tendons were visualized, fully transected, and then repaired using the standard double-row configuration with suture tape and suture anchors. During this repair, the scaffold device (ROTIUM; Atreon Orthopedics, Dublin, OH, USA) was laid directly on the humeral footprint and “sandwiched” between the tendon and its bony footprint, following manufacturer instructions. Standard surgical procedures were followed for closure and the same animal husbandry procedures were followed for the remainder of the study.

Animals were humanely euthanized 12 weeks after the repair surgery (18 weeks after the initial procedure/induction of chronic degeneration). Treated forelimbs were immediately harvested, and the humerus-infraspinatus constructs were isolated and denuded of soft tissues with great care as to not damage the infraspinatus tendon.

### Biomechanical testing

A cross-sectional area (CSA) measurement of all tendons was taken proximal to the insertion using previously validated techniques in which an area micrometer that applied 0.12 MPa of pressure parallel to the cross-section of the tendon.^17^^,^^18^ The humeri were subsequently mounted in polyvinyl chloride sleeves using a strong 2-part hard casting resin (SmoothCast 321; Smooth-On, Macungie, PA, USA) and mounted in a custom fixture attached to a servo-hydraulic load frame (Model 805; MTS Corp., Eden Prairie, MN, USA) which allowed anatomically accurate loading of the tendon.^9^^,^^28^ Prior to biomechanical testing, tendons underwent a preloading phase to normalize viscoelastic effects in which a static preload of 5N was applied for a duration of 2 minutes,^21^^,^^29^^,^^36^ followed by nondestructive stress relaxation testing at the physiologically relevant strain level of 6% (outcomes: peak stress [MPa], peak force [N], equilibrium force [N], equilibrium stress [MPa], and percent relaxation [%]).^19^ Tendon hydration was maintained with physiological saline during the entire preparation and mechanical testing procedure.

### Histological evaluation

Immediately following mechanical testing, samples were bisected through the tendon insertion, fixed in 10% neutral buffered formalin (≥ 7 days), dehydrated in graded solutions of ethanol (Tissue-Tek VIP; Sakura, Torrance, CA, USA), and then cleared with acetone and polymerized into a hardened plastic block (Hard Acrylosin; Dorn and Hart Microedge, Villa Park, IL, USA). Final histology slides were produced in the sagittal plane to display the humeral bone, repair site (ie, enthesis), implant (if applicable), and surrounding tendon/soft tissue. Sections were stained with Sanderson rapid bone stain (which provides differentiation of cells within the section and allows detection of cartilage) and then counterstained with Van Gieson bone stain (allowing the differentiation of collagen and detection of bone [immature woven bone and mature lamellar bone]).

Slides were graded by a board-certified veterinary pathologist using a modified International Organization for Standardization (ISO) 10993/6 scoring rubric to assess the biocompatibility, including cellular response and inflammation and/or foreign body response (Table I). Additionally, a scoring system (previously implemented^27^) based on an adaptation of parameters from the Bonar scoring system was used for specific assessment of tendon histologic characteristics (Table II).

**Table I:** Histological evaluation of tendon-bone tissue sections for inflammatory cell type and response.

Cell type/response	Score				
	0	1	2	3	4
Polymorphonuclear cells	None	Rare, 1-5/HPF∗	5-10/HPF	Heavy infiltrate	Packed
Lymphocytes	None	Rare, 1-5/HPF	5-10/HPF	Heavy infiltrate	Packed
Plasma cells	None	Rare, 1-5/HPF	5-10/HPF	Heavy infiltrate	Packed
Macrophages	None	Rare, 1-5/HPF	5-10/HPF	Heavy infiltrate	Packed
Giant cells	None	Rare, 1-2/HPF	3-5/HPF	Heavy infiltrate	Sheets

∗ HPF, per high powered (400 x) field.

**Table II:** Grading rubric for tendon and tendon-bone interface histological changes.

Tendon changes	Score				
	0	1	2	3	4
Neo-vascularization	*None*	*Minimal*: Few blood vessels (< 3/10x field) randomly distributed throughout tendon	*Mild*: Few vessels (< 5/10x field) uniformly/diffusely distributed throughout tendon	*Moderate*: Moderate numbers of vessels (5-10/10x field) distributed diffusely throughout tendon	*Severe*: Large numbers of vessels (∼ > 10/10x field) distributed diffusely throughout tendon
Tenocyte reactivity/stromal cell proliferation	*None*	*Minimal*: Multifocal increased prominence of stromal cells between parallel collagen bundles, few tenocytes/stromal cells with plump reactive nuclei	*Mild*: Multifocal proliferation of stromal cells with plump nuclei which results in mild separation of collagen bundles	*Moderate:* Diffuse proliferation of stromal cells between collagen bundles, OR multifocal proliferations of stromal cells which markedly disrupt/displace collagen bundles	*Severe*: Diffuse, marked proliferation of stromal cells resulting in a hypercellular appearance of tissue and marked separation of collagen bundles/disruption of normal architecture
Collagen fiber structure and arrangement	*None*: normal compact and parallel collagen fiber alignment	*Minimal:* Multifocal Increased waviness/separation of collagen fibers	*Mild:* Diffuse increased waviness and separation of collagen fibers; mild loss of parallel arrangement	*Moderate:* Marked increased waviness, separation, and loss of collagen fiber structure. Collagen bundles are nonparallel and haphazardly arranged	*Severe:* Collagen fiber degeneration (hyalinization, mucinous degeneration) bundles appear haphazard with complete loss of parallel arrangement

Tendon-Bone Interface	0	1	2	3	4
Enthesis type and histological character	*None:* (Re-establishment) of normal/native direct tendon to bone attachment with a predominance (> 50%) of fibrocartilaginous attachment zones	*Minimal:* Complete attachment of tendon to bone with multifocal organized fibrocartilaginous zones of attachment (comprises < 50% of attachment)	*Mild:* Tendon-bone attachment with small disorganized zones of fibrocartilaginous (direct type) attachment (comprises < 25% of tendon-bone interface)	*Moderate:* Complete tendon-bone attachment composed entirely of fibrous tissue (indirect attachment type)	*Severe:* Partial or complete lack of tendon attachment to bone (no evidence organized tendon attachment, either fibrous or fibrocartilaginous)
Presence of Sharpey-like fibers	0 = Yes	1 = No			

### Statistical analysis

All comparisons between the 2 groups were made using a student’s *t*-test (GraphPad Prism v10.0.0; GraphPad, San Diego, CA, USA). All data passed Anderson-Darling normality test. Sample sizes were chosen to achieve 80% statistical power using results from previous studies^16^^,^^17^^,^^27^ to detect a 25% increase in biomechanical properties (ie, peak stress). Four sheep were assigned to each group.

## Results

### Biomechanical testing

**Figure 2:**
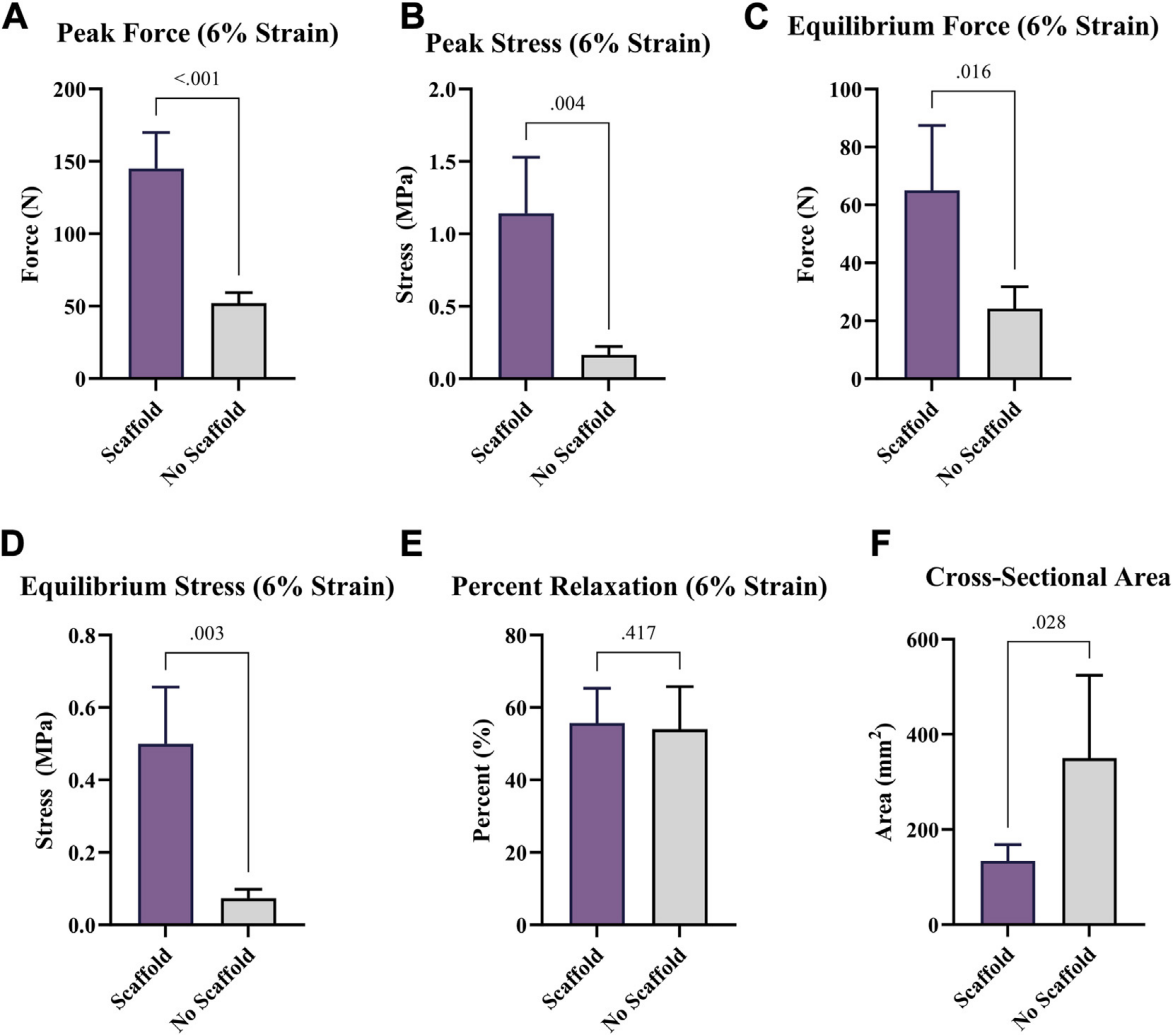
Biomechanical testing results. (**A**) Peak force, (**B**) peak stress, (**C**) percent relaxation, (**D**) equilibrium force, and (**E**) equilibrium stress from the stress-relaxation testing. (**F**) Cross-sectional area of tendon samples. Bar charts illustrate mean ± std. dev. for each group. *Std. dev.*, standard deviation.

The scaffold group exhibited significantly improved biomechanical properties: increased peak force (279% increase, *P* < .001, Fig. 2, *A*), increased peak stress (686% increase, *P* = .004, Fig. 2, *B*), increased equilibrium force (269% increase, *P* = .016, Fig. 2, *C*), and increased equilibrium stress (682% increase, *P* = .003, Fig. 2, *D*). No significant difference was found between groups in percent relaxation (*P* = .42, Fig. 2, *E*). The scaffold group exhibited a significantly decreased CSA (61.6% decrease, *P* = .028, Fig. 2, *F*), similar to what has been reported for healthy/uninjured tendons previously.^16^

### Quantitative histological evaluation

**Figure 3:**
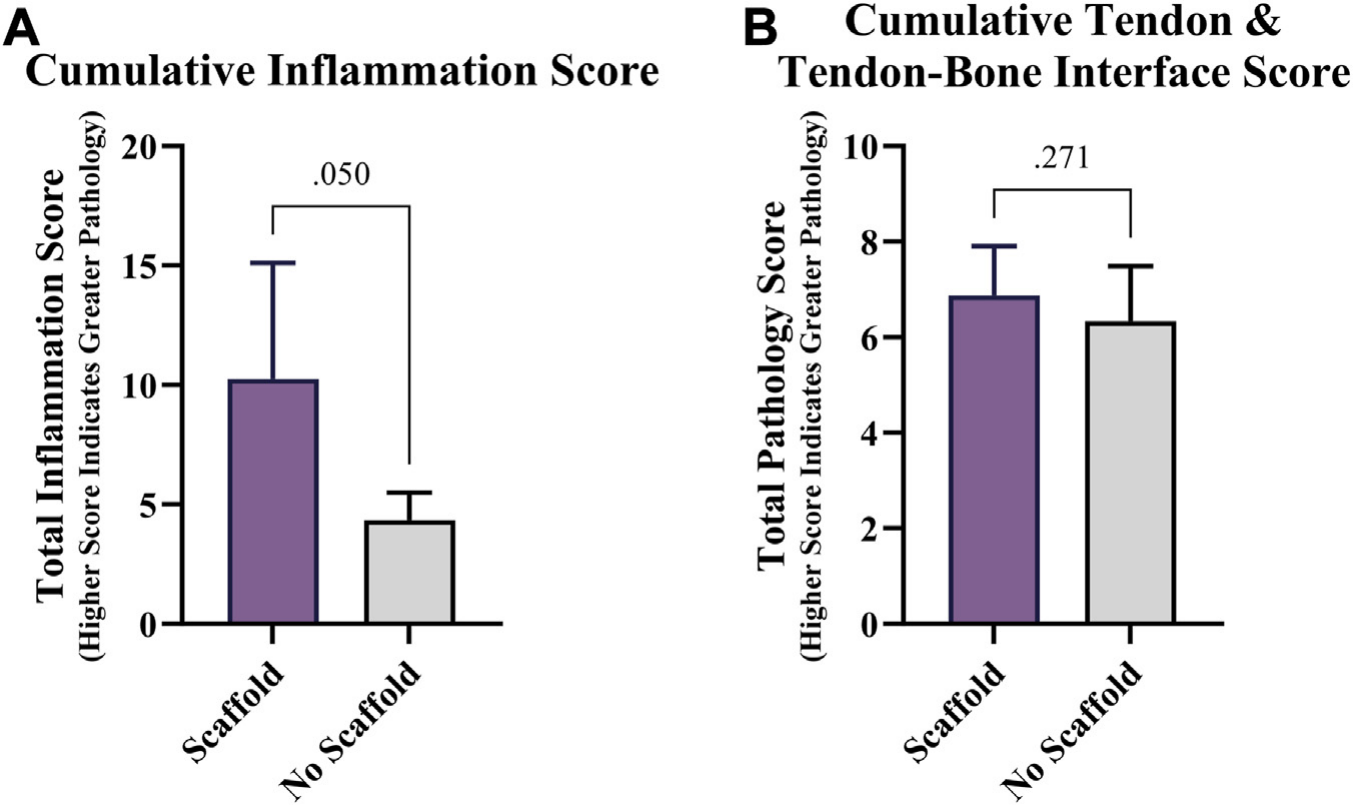
Semi-quantitative histopathology scoring of tendon samples. (**A**) Cumulative inflammation score indicating the increased presence of polymorphonuclear cells, lymphocytes, plasma cells, macrophages, and giant cells. (**B**) Cumulative tendon and tendon-bone interface score, indicating the neo-vascularization, tenocyte reactivity/stromal cell proliferation, collagen fiber structure and arrangement, enthesis type/histological character, and the presence of Sharpey-like fibers.

As determined by quantification of polymorphonuclear cells, lymphocytes, plasma cells, macrophages, and giant cells, the scaffold group exhibited significantly increased cumulative inflammation score (*P* = .050, Fig. 3, *A*). A significant difference in cumulative tendon and tendon-bone interface score was not found (*P* = .271, Fig. 3, *B*).

**Figure 4:**
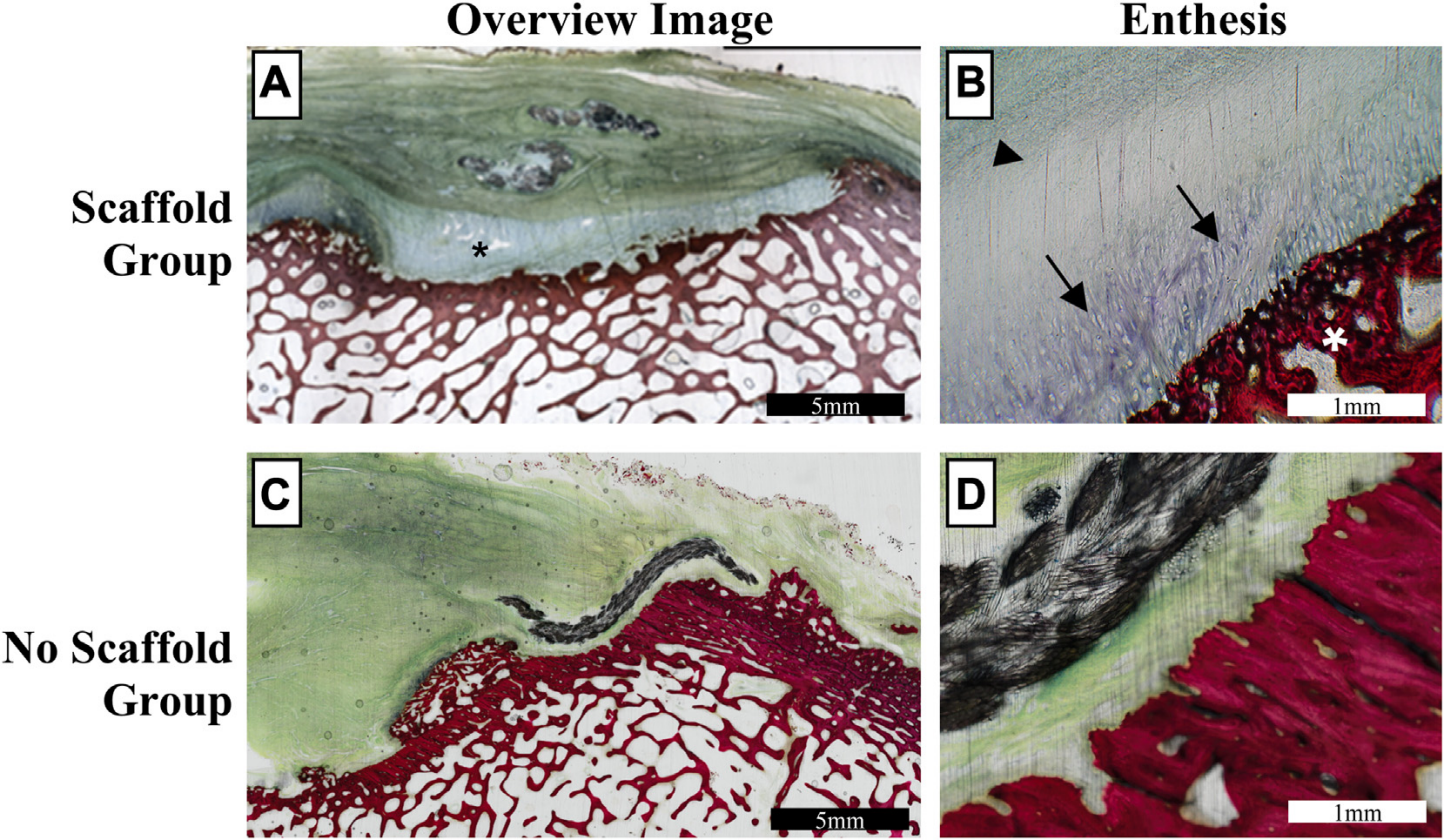
Representative histology slide images of the scaffold and no scaffold groups. (**A**) 2x magnification image of a representative scaffold sample, demonstrating the scaffold device in place in the inlay position (*asterisk*). (**B**) 10x magnification image of enthesis region of same scaffold sample, segmentally demonstrating partial recapitulation of a more direct type (fibrocartilaginous) enthesis, characterized by transition from tendon fibrous tissue (*arrowhead*) to fibrocartilage (*arrows*) which attach to bone (*white asterisk*). (**C**) 2× magnification image of a representative no scaffold sample. (**D**) 10× magnification image of enthesis region of same no scaffold sample, demonstrating lack of direct type enthesis.

### Descriptive histological evaluation

Enthesis changes in the scaffold group were characterized by marked disorganization and complete loss of normal enthesis architecture, with replacement by abundant amounts of dense reactive fibrous connective tissue, as well as frequent reactive new woven bone being laid down along the humeral footprint (Fig. 4). Approximately half of these samples exhibited focal attempts at re-establishment of a native/direct-type fibrocartilaginous enthesis. These were characterized by a very thin zone of calcified fibrocartilage, which formed the transition from the reactive fibrous tissue to new woven bone along the humeral footprint (occupying approximately 10%-20% of the surface area of the humeral footprint). Some Sharpey-like fibers would be observed in these regions (present in 2 of 4 samples).

Histological changes for the “no scaffold” group demonstrated changes which were in-line (similar in character and degree) to those observed for the scaffold group, characterized by a predominately fibrous connective tissue (indirect-type) enthesis, with minimal to no re-establishment of a fibrocartilaginous enthesis, and typically no visible Sharpey fibers (present in 2 of 3 samples). Additionally, the tendons of these animals had minimal to mild tenocyte reactivity and collagen fiber disarray, with minimal to mild chronic mononuclear inflammation.

## Discussion

In this study, we investigated the biomechanical and histological impacts of augmenting rotator cuff repairs with the biphasic nanofiber scaffold in a model of chronically degenerated tendon tissue. Our key findings reveal that the use of this scaffold leads to a significant enhancement in the biomechanical strength of the repair constructs. Additionally, histological analysis indicated the scaffold did not impede re-establishment of the fibrocartilaginous insertion at the tendon-bone interface. These results suggest that the scaffold could play a pivotal role in re-establishing the native enthesis and improving the outcomes of rotator cuff repairs, particularly in challenging cases involving chronically degenerated tendons.

Previous studies have characterized the improvements to biomechanical strength, structural improvements, and healing outcomes of rotator cuff repairs augmented with this nanofiber interpositional scaffold. Romeo et al demonstrated significant improvements in the ultimate strength of rotator cuff repairs in an acute repair sheep model, highlighting the scaffold’s ability to replicate native Sharpey-like fibers and enhance repair strength.^27^ Seetharam et al in 2022 documented the scaffold’s efficacy in human rotator cuff repairs, reporting a notable 91% tendon healing rate, as evidenced by magnetic resonance imaging, and marked improvements in functional outcomes.^30^ These studies, underpinning the biomechanical and clinical advantages of the scaffold, corroborate the findings of our research, which extends these benefits to rotator cuff repairs in chronically degenerated tendon tissue. Together, these investigations fortify the potential of the nanofiber scaffold as a transformative tool in enhancing the outcomes of rotator cuff repairs across various clinical scenarios by affecting the biological milieu through the scaffold degradants.

In the use of this scaffold for rotator cuff repair, it is typically positioned at the interface between the tendon and bone at the repair site. The scaffold is secured using 1 or 2 suture anchors, depending on the repair technique (single or double row). The scaffold is designed to integrate into the surgical procedure without adding significant time and is compatible with all suture anchors, as it is simply secured by routing the suture tape through a hole in the scaffold. The scaffold’s interpositional placement beneath the tendon allows for effective tendon-bone integration, while its bioresorbable nature supports tissue healing without the need for additional disposables. Platelet-rich plasma or other biologics can be applied to enhance the regenerative effects, although their use is tailored to individual patient needs and no data have yet been generated to demonstrate efficacy when used with this product.

These positive improvements in biomechanical properties attained with augmentation of this scaffold are noteworthy in comparison to other rotator cuff repair augmentation scaffolds, which focus on biomechanical augmentation but have not demonstrated significant improvements in biomechanical properties. While other commercially available products have demonstrated improvements in surgical outcomes, to date no studies have demonstrated improved repair construct strength using these devices. However, it is crucial to remember that clinical outcomes, although important, are only part of the success metric. Many of the studies demonstrating “successful” outcomes using these products do not include a control group, thus precluding the determination whether the scaffold generated improved outcomes.^22^^,^^34^ Arguably, the most critical measure of a device’s efficacy in rotator cuff repair is early healing and the improvement in the strength of the healed tissue (ie, restoration of the native enthesis), which directly impacts the long-term success and durability of the repair. For this reason, the data presented herein support the use of this nanofiber interpositional scaffold for augmentation of chronic rotator cuff repair.

The observation that the scaffold group achieved a higher peak force despite having a lower CSA is particularly noteworthy. This finding challenges the common perception that tendon thickening, as marketed by many recent products, is inherently beneficial.

This nanofiber scaffold was specifically engineered to re-establish the native enthesis through its unique architecture and chemical composition, focusing on enhancing the quality of the tendon-bone interface rather than increasing tendon mid-body thickness. This design allows the scaffold to distribute mechanical loads more effectively, leading to significant improvements in biomechanical strength. The result is a repair that is both physiologically appropriate and mechanically robust, just like the native enthesis. As shown in Figure 3, the scaffold group demonstrated higher markers of inflammation compared to the control group. This is due to the degradation products of the scaffold which are involved in the modulation of macrophage activity, shifting them toward a pro-healing M2 phenotype, which is essential for tissue repair and regeneration.^13^^,^^31^ These bioactive degradants not only ensure the scaffold’s resorption but also actively participate in the healing process, which is a critical component in chronic tendon injuries with little to no vascular supply. Without inflammation, there will be no active remodeling which is demonstrated by the poor biomechanical strength in the control group.

This study, while providing important insights, has certain limitations that should be acknowledged. First, the sample size was relatively small, which may limit the generalizability of the findings. A post hoc power analysis revealed this study has an 84.3% power for the peak stress outcome parameter, which is generally considered adequate. Additionally, the study was conducted in a controlled laboratory setting using a preclinical animal model of chronically degenerated cuff tissue as it is not plausible to generate biomechanical testing data using human patients. Furthermore, there was only one timepoint used in this work, preventing assessment of temporal healing. Future research should aim to address these limitations and further validate clinical improvements, ideally with large, diverse patient cohorts and extended follow-up periods to comprehensively evaluate the long-term efficacy and safety of the scaffold in a real-world clinical setting.

## Conclusion

The ROTIUM Bioresorbable Wick offers a significant advancement in rotator cuff repair by enhancing biomechanical properties at the repair site, as evidenced by a substantial increase in peak force and a CSA comparable to healthy tendons. Despite limitations in sample size and study duration, these findings indicate that the scaffold could potentially reduce failure rates and improve long-term outcomes in rotator cuff surgeries. Further research with larger, diverse human patient cohorts is necessary to validate these promising results and fully realize its clinical benefits.

## Disclaimers:

Funding: This study was funded by a research grant from Atreon.

Conflicts of interest: Jed Johnson is a paid employee of Atreon. The other authors, their immediate families, and any research foundation with which they are affiliated have not received any financial payments or other benefits from any commercial entity related to the subject of this article.
